# Supplementary material for: Temporal, spatial and gender-based dietary differences in middle period San Pedro de Atacama, Chile: A model-based approach
Source: PLoS One. 2021 May 25;16(5):e0252051. doi: 10.1371/journal.pone.0252051 (PMC8148334; doi:10.1371/journal.pone.0252051)
Supplement: Attachment — Submitted filename: Response to reviewers.docx [file pone.0252051.s002.docx]

Please see responses to individual reviewer comments/suggestions below, in red.

PONE-D-20-41004

Temporal, Spatial, and Gender-based Dietary Differences in Middle Period San Pedro de Atacama, Chile: A Model-based Approach.

Please include the following items when submitting your revised manuscript:

A rebuttal letter that responds to each point raised by the academic editor and reviewer(s). You should upload this letter as a separate file labeled 'Response to Reviewers'.

Done.

A marked-up copy of your manuscript that highlights changes made to the original version. You should upload this as a separate file labeled 'Revised Manuscript with Track Changes'.

Done

An unmarked version of your revised paper without tracked changes. You should upload this as a separate file labeled 'Manuscript'.

Done

If applicable, we recommend that you deposit your laboratory protocols in protocols.io to enhance the reproducibility of your results. Protocols.io assigns your protocol its own identifier (DOI) so that it can be cited independently in the future. For instructions see: <http://journals.plos.org/plosone/s/submission-guidelines#loc-laboratory-protocols>

We will look into the possibility of depositing our laboratory protocols in protocols.io.

Journal Requirements:

When submitting your revision, we need you to address these additional requirements.

1. Please ensure that your manuscript meets PLOS ONE's style requirements, including those for file naming. The PLOS ONE style templates can be found at

https://journals.plos.org/plosone/s/file?id=wjVg/PLOSOne_formatting_sample_main_body.pdf and

https://journals.plos.org/plosone/s/file?id=ba62/PLOSOne_formatting_sample_title_authors_affiliations.pdf

Manuscript (text and references) has been reformatted to PLoS One style requirements.

2. Please ensure that you include a title page within your main document. You should list all authors and all affiliations as per our author instructions and clearly indicate the corresponding author.

Completed as requested.

3. Please note that in order to use the direct billing option the corresponding author must be affiliated with the chosen institute. Please either amend your manuscript to change the affiliation or corresponding author, or email us at plosone@plos.org with a request to remove this option.

Corresponding author (Pestle) noted.

4. Please include your tables as part of your main manuscript and remove the individual files. Please note that supplementary tables (should remain/ be uploaded) as separate "supporting information" files

All tables have been added as Microsoft Excel objects in the main manuscript.

5. We note that Figure 1 in your submission contains map/satellite images which may be copyrighted. All PLOS content is published under the Creative Commons Attribution License (CC BY 4.0), which means that the manuscript, images, and Supporting Information files will be freely available online, and any third party is permitted to access, download, copy, distribute, and use these materials in any way, even commercially, with proper attribution. For these reasons, we cannot publish previously copyrighted maps or satellite images created using proprietary data, such as Google software (Google Maps, Street View, and Earth). For more information, see our copyright guidelines: http://journals.plos.org/plosone/s/licenses-and-copyright.

We require you to either (1) present written permission from the copyright holder to publish these figures specifically under the CC BY 4.0 license, or (2) remove the figures from your submission:

We have modified the Figure 1 basemap layer to use imagery from the USGS National Map Viewer, which is in the public domain.

1. the manuscript is not formatted according to Plos One instructions, nor the text nor the reference section. Please, follow in detail the Plos One guidelines for authors.

Manuscript (text and references) has been reformatted to PLoS One style requirements.

2. There is a substantial lack of information about the archaeology of the graveyards. From which kind of funerary assemblages is the skeletal record coming from? What about the representativeness, in term of demographic profiles, of the single necropoles. Is the skeletal record a reasonable proxy of the ayllus society? Are the skeletal samples from single ayllus comparable? We perfectly know how the Osteological Paradox and the always non-random selection of the funerary record limit our biocultural reconstructions. The authors have to supply a concise but exhaustive description of the funerary contexts and if it is homogeneous through time and space. A better description of the "incredibly well preserved and contextualized human skeletons" should be made available for the reader.

A new section (skeletal collections) has been added to Material and Methods to offer more information on the skeletal samples provenience and representativeness.

3.Similarly, the bioarchaeological record is not enough reported, and few lines of the basic methods used for sex and age at death determination should be provided. Are all the individuals fully adult ones? so subadults? If " we recently completed intensive bioarchaeological and biogeochemical analysis of a large set (over 600 individuals) of incredibly well preserved and contextualized human skeletons from the Middle Period of the San Pedro oases" what was the criterion used to select the 288 individuals?

See the new section in Material and Methods for details on bioarchaeological methods, and the added information in the Sampling section that clarifies the selection criteria for isotopic samples.

4. The authors use the MDS for some data analysis, please specify more in detail which flavour and the R packege used.

Details were added to M&M about the MDS.

5. " while inequality of some sort is inherent in all human societies", page 2, is a too deterministic and maybe not necessary sentence

This sentence has been modified to remove the phrase noted.

6. page 32 " A sufficient number of sexed individuals were available from only four ayllus: Coyo, Larache, Quitor, and Solcor." then in page 32 "The final iteration of this sex-based analysis combined ayllu and sex, forming twelve groups..", then in figure 16 5 male groups and 6 female, same in figure 17. According to Table 1 the sexed individuals are

Conde Duque 7

Coyo 74

Larache 18

Quitor 31

Solcor 54

Tchecar 15

18 individuals from Larache aren’t much more than the 15 in Tchecar. I suggest to fix this point and to use (if possible) all the subsample or to better justify the exclusions.

We have attempted to address in the text both the reasons for the exclusion of Tchecar and our decision to justify the focus on Larache.

Finally, a table in the text summarizing the number of individuals by sex and age at death for the six sub-samples could be useful.

A new Table (Table 11) is now in the text with sex-by-ayllu data. Age data were not supplied for reasons discussed below (lack of subadults and imprecision of adult age estimates).

Reviewers' comments:

Reviewer #1: Very well-written and analyzed paper with a lot of information packed into a small space. Great sample size and background. Very nice introduction and convincing narrative.

Here are the few questions that arose as I read through it:

1. The paper explores the existence of inequalities in San Pedro de Atacama during the Middle Period. Are scholars directly addressing this question by using other bioarchaeological methods?

This is detailed in the new section “Skeletal Collections” in Materials and Methods.

2. Three main potential vectors of difference (time, context and sex) are explored. Why age is not explored as a potential vector of difference?

The reasons for omission of age as a vector or difference are noted in the Research questions stcion. The sample of 257 well-preserved individuals includes only 15 subadults (5.8%), and as many of the adult individuals are represented only by cranial remains, precise age determination was not possible.

3. Model-based paleodietary reconstruction is an interesting approach, and can help address differences among and within populations. However, many archaeologists may be unaware of the complexities and pitfalls of stable isotope mixing models. I do not think it is outside of the scope of this research to briefly discuss the idea that mixing models in paleodietary reconstructions can hardly provide an exact calculation of food group contributions, despite their potential to provide new lines of enquiry.

A discussion of the limitations of model-based approaches to paleodietary estimation has been added as requested/.

Reviewer #2: This is an excellent study that expands significantly beyond the approach usually used in archaeology by using a large data set and by applying statistical analysis to the results. My main concern with this paper is that there is almost no mention of where the skeletal and dental material comes from. If these samples are from archaeological sites, please provide some information about them. It is also unclear how the samples have been classified into ayllu.

See new section on Skeletal Collections that offers the information requested.
